# Preclinical Evaluation of a Microneedle-Delivered Adjuvanted Microparticulate Gonorrhea Vaccine in Non-Human Primates

**DOI:** 10.3390/vaccines14090827

**Published:** 2026-09-20

**Authors:** Amarae Ferguson, Yashkumar Harsoda, Dedeepya Pasupuleti, Tanisha Manoj Arte, Priyal Bagwe, Mahek Gulani, Emmanuel Adediran, Mohammad Uddin, Nikolai Petrovsky, Martin J. D’Souza, Susu M. Zughaier

**Affiliations:** 1 Vaccine Nanotechnology Laboratory, Center for Drug Delivery Research, College of Pharmacy, Mercer University, Atlanta, GA 30341, USA; amarae.ferguson@live.mercer.edu (A.F.); yashkumar.pankajbhai.harsoda@live.mercer.edu (Y.H.); dedeepya.pasupuleti@live.mercer.edu (D.P.); tanisha.manoj.arte@live.mercer.edu (T.M.A.); priyal.bagwe@live.mercer.edu (P.B.); mahekanil.gulani@live.mercer.edu (M.G.); eadediran@larkin.edu (E.A.); uddin_mn@live.mercer.edu (M.U.); 2 Australian Respiratory and Sleep Medicine Institute, Adelaide, SA 5042, Australia; nikolai.petrovsky@vaxine.net; 3 Vaxine Pty Ltd., Adelaide, SA 5046, Australia; 4 College of Medicine, QU Health, Qatar University, Doha P.O. Box 2731, Qatar

**Keywords:** *Neisseria gonorrhoeae*, pre-clinical, primates, vaccine, adjuvants, microparticles

## Abstract

**Background/Objectives:** Gonorrhea remains one of the most prevalent sexually transmitted infections globally, with an estimated 82–87 million new infections annually and no licensed vaccine available. Antigenic variability and the lack of durable protective immunity following natural infection have been major obstacles to vaccine development. We previously developed an adjuvanted, formalin-inactivated whole-cell gonococcal microparticle (GC-MP) vaccine delivered by dissolving microneedles and demonstrated that it induced functional antibodies and enhanced bacterial clearance in murine models. Here, we evaluated this GC-MP microneedle platform in *Rhesus macaques* to provide proof-of-concept data in a relevant large-animal model and to compare different adjuvants. **Methods:** *Rhesus macaques* were allocated into five groups and vaccinated, and serum antigen-specific IgG and IgA responses were quantified by ELISA, with functional activity assessed by serum bactericidal assay. In parallel, the potency of delta-inulin-, aluminum-phosphate-, or aluminum-hydroxide-adjuvanted *N. gonorrhoeae*-loaded microparticles to activate dendritic cells was assessed in vitro via changes in surface expression of MHC I, MHC II, CD40, and CD80. **Results:** The serum from vaccinated primates demonstrated elevated gonococcal-specific IgG and IgA responses compared with naïve controls and measurable bactericidal activity against live, homologous *N. gonorrhoeae* strains. All adjuvanted formulations increased dendritic cell expression of MHC I, MHC II, CD40, and CD80. **Conclusions:** These findings indicate that an adjuvanted whole-cell inactivated gonorrhea vaccine delivered transdermally via microneedles elicits clearly elevated, relative to controls, functional antibody responses, supporting its advancement toward clinical evaluation.

## 1. Introduction

Gonorrhea is a highly prevalent sexually transmitted infection caused by the Gram-negative bacterium *Neisseria gonorrhoeae*, with tens of millions of new cases estimated to occur globally each year [1]. In many countries, including the United States, gonorrhea ranks among the most reported notifiable infections, with the highest incidence typically seen in adolescents and young adults, and a disproportionate burden in marginalized populations. If left untreated, gonococcal infection can lead to serious reproductive and neonatal sequelae such as pelvic inflammatory disease, ectopic pregnancy, infertility, and adverse pregnancy outcomes, as well as increased susceptibility to HIV acquisition and transmission [2,3]. The public health impact of gonorrhea is further compounded by the rapid and ongoing emergence of antimicrobial resistance in *N. gonorrhoeae*, which has successively eroded the efficacy of multiple antibiotic classes and left extended-spectrum cephalosporins as the last reliable first-line therapy in many settings [4,5].

Despite its global burden and the growing threat of untreatable infection, there is currently no licensed vaccine against gonorrhea [6]. Several historical vaccine candidates have been evaluated in humans, but none have demonstrated convincing protective efficacy. Early efforts using crude whole-cell preparations, including a field trial in a highly endemic population in northern Canada, caused high reactogenicity but ultimately failed to demonstrate meaningful protection, likely due to suboptimal antigen presentation [7]. More targeted approaches, such as vaccines based on individual surface components like pilus (Pil) proteins, also proved unsuccessful, in part because *N. gonorrhoeae* exhibits extensive antigenic and phase variation in key surface antigens, allowing the bacterium to evade highly focused host or vaccine responses [8]. Additional challenges include *N. gonorrhoeae*’s ability to suppress and modulate host immunity, the lack of sterilizing natural immunity following infection, and the difficulty of identifying conserved, functionally important antigens that are broadly expressed across diverse strains [9].

Dissolving microneedle patches are minimally invasive transdermal delivery systems comprising arrays of micron-scale projections fabricated from water-soluble, biocompatible materials. Following application, the microneedles penetrate the stratum corneum and dissolve, releasing the incorporated vaccine components into the epidermal and dermal compartments without leaving residual sharp waste. This delivery route is particularly attractive for vaccination because the skin contains abundant antigen-presenting cells, including epidermal Langerhans cells and dermal dendritic cells, that can capture antigen and initiate adaptive immune responses [10]. In our formulation, hyaluronic acid serves as the water-soluble needle-forming matrix containing the vaccine microparticles and adjuvant, whereas PVA forms a supporting backing layer that provides structural integrity during patch handling and application. Trehalose, a nonreducing disaccharide, is included as a stabilizing excipient to improve mechanical strength, improve dissolution, and preserve antigenic integrity [11]. Dissolving microneedle patches have generated protective immune responses in preclinical influenza-vaccine studies and have advanced to clinical evaluation; notably, a randomized phase I trial demonstrated that influenza vaccination by dissolving microneedle patch was well tolerated and elicited robust antibody responses [12,13]. These findings support the translational potential of dissolving microneedles as a vaccine-delivery platform. However, their application to an adjuvanted whole-cell gonococcal microparticle vaccine, particularly in a non-human-primate model, has not been extensively explored.

In recent years, advances in antigen discovery, adjuvant technology, and delivery platforms, along with epidemiological signals of cross-protection from related meningococcal vaccines, have renewed interest in gonorrhea vaccine development [14]. Building on prior work demonstrating that an adjuvanted whole-cell inactivated *N. gonorrhoeae* microparticulate vaccine delivered transdermally via a dissolving microneedle “skin patch” can elicit robust humoral and functional responses and confer protection in murine models [15], there is a critical need to evaluate this platform in non-human primates to more closely approximate human immunobiology. One aspect that has been poorly explored in the past is the potential role of different adjuvants to enhance the responses of vaccines delivered via a microneedle patch. This has been driven by the presumption that the high density of Langerhans cells and other dendritic-like cells in the epidermis obviates the need for an adjuvant when delivering antigens through the skin. To the contrary, we have previously shown that the inclusion of traditional adjuvants, such as aluminum salts, in dissolvable microneedle patches further enhances vaccine efficacy [16]. Delta-inulin is a semicrystalline particulate form of inulin, a naturally occurring plant-derived fructan composed predominantly of β-D-(2→1)-linked fructofuranose units with a terminal glucose residue. Soluble inulin exhibits little adjuvant activity, whereas conversion to the relatively insoluble delta crystalline polymorph produces particles with immunopotentiating properties. Advax^®^ is a standardized particulate adjuvant manufactured from delta-inulin with controlled physicochemical characteristics [17]. Unlike aluminum adjuvants, delta-inulin does not require antigen adsorption and is reported to enhance both humoral and cellular immunity without relying primarily on Toll-like receptor signaling or strong inflammasome-mediated inflammation. Recent studies have identified DC-SIGN as a cellular receptor for delta-inulin, providing a potential mechanism for its interaction with dendritic cells and enhancement of antigen presentation [18]. Advax has been evaluated in liquid vaccine formulations in clinical studies of seasonal and pandemic influenza and hepatitis B, as well as allergen immunotherapy, where it was generally well tolerated and showed immunopotentiating or antigen-sparing activity [19]. While delta-inulin adjuvant has been extensively validated for its ability to enhance vaccine efficacy in animal models and humans as part of liquid vaccine formulations administered intramuscularly, it has not previously been tested when incorporated into a dissolving microneedle patch.

In this proof-of-concept study, we evaluated the systemic immunogenicity of whole-cell inactivated gonococcal microparticle vaccines formulated with delta-inulin, aluminum phosphate, or aluminum hydroxide and delivered using dissolving microneedles in *Rhesus macaques*, as assessed by antigen-specific serum IgG and IgA responses and serum bactericidal activity.

## 2. Materials and Methods

### 2.1. Preparation of Gonococcal Agar Plate

Difco^TM^ GC Medium Base (36 g) (BD Difco, Becton Dickinson, Franklin Lakes, NJ, USA) was mixed into 1 L of DI water and autoclaved. Then, 10 mL of D-glucose and 1 mL of iron were added to the mixture, which was gently mixed. The mixture was poured into Petri dishes and left to cool and solidify as previously described [15].

### 2.2. Preparation of Neisseria gonorrhoeae Vaccine Antigen

As we previously described, the desired strain of *N. gonorrhoeae* was first streaked on a Gonococcal Base (GCB) agar containing specific supplements necessary for gonococcal proliferation [15,20]. The bacteria on the agar were then placed in an incubator at 37 °C with 5.0% CO_2_. The colonies would then grow within 24 to 48 h. The freshly grown colonies were used to inoculate flasks containing 500 mL of GC broth supplemented with 5 mL of D-glucose and 500 μL of iron with sodium bicarbonate (0.043%). The flasks were then placed in an incubator to shake at 100 rpm for 6 h. 10% formalin (*v*/*v*) was added to stop gonococcal growth, and the mixture was then incubated overnight while gently mixing at room temperature. The formalin-inactivated gonococcal pellets were collected by centrifugation at 5000 RCF for 15 min at 4 °C. The harvested pellets were washed thrice with sterile PBS (Sigma-Aldrich, St. Louis, MO, USA) and centrifuged as described above. The final collected pellets were pooled, thoroughly vortexed, and stored as a dense suspension at −80 °C.

### 2.3. Evaluation of Particle Size Distribution, Polydispersity Index, and Zeta Potential Measurement

The particle formulation characteristics were evaluated using a Malvern Zetasizer Nano ZS (Malvern Instruments, Malvern, UK). To determine particle size and PDI, 2 mg of particles were suspended in 1 mL of deionized water, vortexed for 1 min, and then measured. For the Zeta potential, 5 µg of microparticles were suspended in 1 mL of deionized water and transferred to a Zeta potential measurement cuvette, and the Zeta potential was measured. All values were measured in triplicate.

### 2.4. Preparation of Dissolving Microneedles

The microneedle preparation process was previously developed in our laboratory [15,20,21,22]. The formulation for the microneedle involved adding 25 μL of DI water, 5% trehalose (Sigma Aldrich, St. Louis, MO, USA), 950 μg of antigen (encapsulated fixed Gonococcal microparticle (GC-MP)), 450 μg of Aluminum hydroxide MPs, 450 μg of Aluminum Phosphate MPs, or 450 μg Delta Inulin MPs, and, lastly, 10% hyaluronic acid (Lifecore Biomedical, Chaska, MN, USA) into a 0.65 mL tube for one microneedle. The components were thoroughly mixed to form a homogeneous viscous suspension. Subsequently, 25 mg of this mixture was dispensed into each premade microneedle mold (Micropoint Technologies, Singapore) and centrifuged at 4000 rpm at 15 °C for 15 min. After centrifugation, the mold was placed into a desiccator overnight. When dry, polyvinyl alcohol (PVA) (Sigma Aldrich, St. Louis, MO, USA) and water were added to form the backing layer, which was then placed in the desiccator for 6 h. The 450 µg adjuvant dose was selected as a fixed, formulation-compatible dose that could be incorporated with 950 µg of GC-MP into the dissolving microneedle matrix while maintaining the patch’s gross structural integrity. The same nominal mass was used for delta-inulin, aluminum phosphate, and aluminum hydroxide to facilitate an exploratory comparison among the three adjuvanted formulations. This dose was not established through a formal dose-ranging study and therefore represents a proof-of-concept formulation dose rather than an optimized biological dose.

### 2.5. Scanning Electron Microscopy of Microparticles and Microneedles

Scanning electron microscopy was used to characterize the microparticle size distribution and surface features, as well as the microneedle patch’s needle size and surface features. The microparticles and microneedle patch were affixed to metal stubs with double-sided adhesive tape, then sputter-coated under vacuum with a thin gold layer (approximately 100–150 Å). The coated samples were subsequently imaged using a Phenome benchtop scanning electron microscope (Nanoscience Instruments, Phoenix, AZ, USA).

### 2.6. Immunization of Monkeys

**Figure 1:**
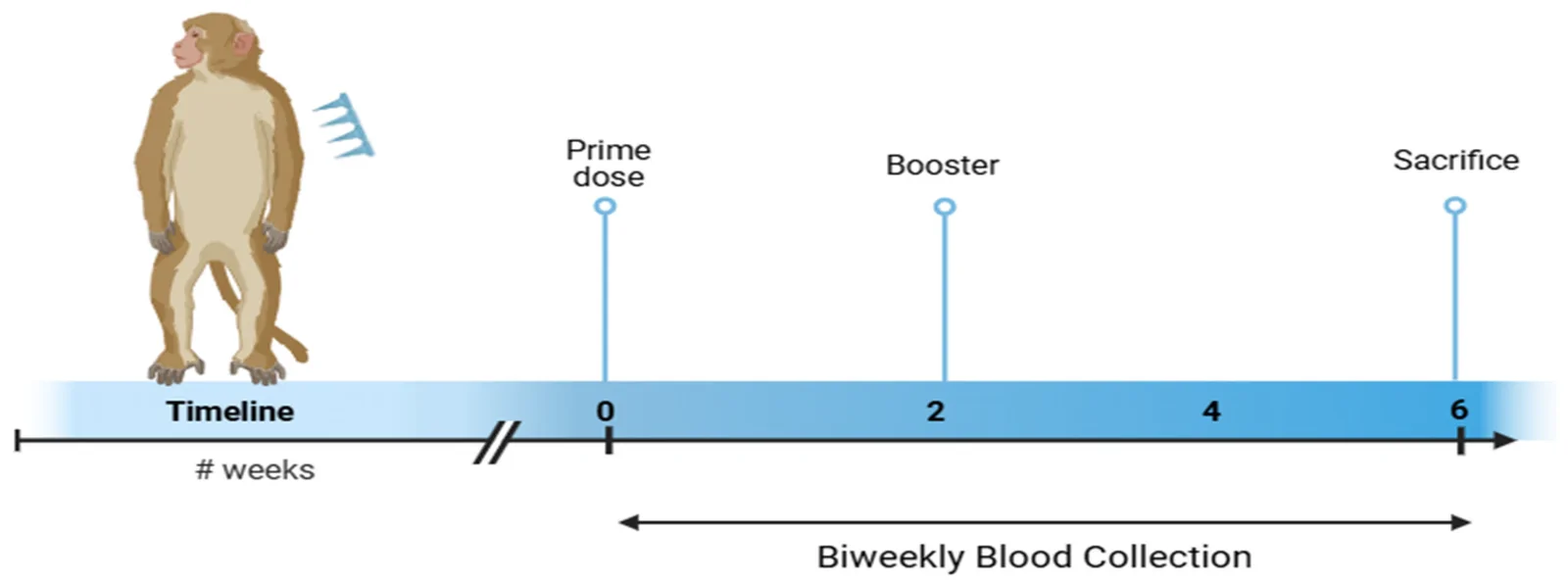
In vivo study timeline: The vaccine formulation’s efficacy was assessed in vivo using *Rhesus macaques*. The monkeys were immunized with a prime dose at week 0, followed by a booster dose at week 2. The monkeys were euthanized at week 6.

*Rhesus macaques* were used as a model to assess the immunogenicity of the gonorrhea microneedle vaccine. Fifteen animals were randomly assigned to five groups (naïve, blank HSA microneedle, GC-MP + delta inulin, GC-MP + aluminum phosphate, GC-MP + aluminum hydroxide), with *n* = 3 monkeys per group (Table 1). One day before being vaccinated, the monkeys had the hair on their upper arm shaved to prepare them for the transdermal delivery of microparticles. The monkeys were immunized the next day by holding down the microneedle on their exposed skin for 10 min, following the schedule below. The 10 min application time was chosen based on previous microneedle studies using the same hyaluronic acid matrix and pilot observations showing that the microneedles became visibly blunted and largely dissolved within this period, consistent with adequate antigen release into the skin. Blood samples were collected every two weeks to assess vaccine immunogenicity (Figure 1). At week 6, animals were humanely euthanized, and terminal blood samples were collected for analysis.

All non-human primate procedures were conducted at Alpha Genesis, Inc. (Yemassee, SC, USA). The study protocol entitled ‘NHP Vaccine Comparison Study’ (AUP 20–08(23)) was reviewed and approved by the Alpha Genesis, Inc. Institutional Animal Care and Use Committee (IACUC) on 5 May 2023, and all procedures complied with institutional and national guidelines for the care and use of laboratory animals. Peripheral blood was collected at weeks 0, 2, 4, and 6, yielding approximately 25 mL of serum from each rhesus macaque over the course of the study; week 6 represented the terminal time point, at which animals were euthanized following the final blood draw in accordance with approved humane endpoints.

### 2.7. Measurement of Antigen-Specific Antibodies Induced in Immunized Primates (Rhesus macaques) Using Indirect ELISA

First, formalin-fixed whole-cell *N. gonorrhoeae* (vaccine antigen, 200 ng/well) was coated on poly-L-lysine-coated high-binding 96-well plates and left overnight at 4 °C. Then, the plates were decanted and blocked for 2 h at 37 °C with 100 μL/well of 2% nonfat milk (ChemCruz, Santa Cruz Biotechnology, Dallas, TX, USA) containing 0.05% Tween-20 (Biorad, Hercules, CA, USA). The plates were washed 2 times, then treated with monkey serum collected before each vaccination, diluted 1:320, and incubated overnight at 4 °C. Then the plates were washed 2 times, and goat anti-monkey IgG Fc secondary antibody with an HRP tag (1:2000) (R&D Systems, Minneapolis, MN, USA), or goat anti-monkey IgA Fc Antibody with an HRP tag (1:2000) (Vector Laboratories, Inc., Newark, CA, USA), was added and incubated for 1 h at 37 °C. The plates were then washed 2 times, and 100 μL TMB substrate reagent (3,3′,5,5″ -tetramethylbenzidine) was added to each well (BD Bioscience, San Jose, CA, USA). Finally, the reaction was stopped with 100 μL of 2N sulfuric acid, and the absorbance was read at 450 nm using a BioTek^®^ Synergy H1 microplate reader (Winooski, VT, USA). Antigen-specific IgG and IgA responses were quantified as optical density (OD) at 450 nm at a fixed serum dilution of 1:320. This single-dilution OD was used as a comparative measure of response magnitude between groups and over time.

### 2.8. Assessment of the Serum Bactericidal Activity

Serum samples obtained from monkeys at week 6 after immunization were tested for bactericidal activity against the CDCF62 strain of live *Neisseria gonorrhoeae*. Monkey sera were first heat-treated at 56 °C for 30 min to inactivate endogenous complement. The heat-inactivated sera were then serially diluted (1:10–1:100,000) in PBS in 96-well plates. Gonococci (CDCF62) were cultured on supplemented GCB agar, harvested, and resuspended in GCB broth to a final density of 2000 CFU/mL. A 40 µL aliquot of this bacterial suspension was added to each well containing diluted sera, gently mixed, and incubated for 15 min at 37 °C in 5% CO_2_. Pooled human serum, serving as an exogenous complement source, was then added to each well, and plates were incubated for an additional 45 min at 37 °C. To determine bacterial survival, 100 µL from each reaction well was plated onto GCB agar and incubated for 24–48 h at 37 °C with 5% CO_2_, after which surviving colonies were enumerated. Percent survival was calculated for each condition as the ratio of the CFU count in serum + complement wells to the mean CFU count in complement-only control wells (bacteria incubated with pooled normal human serum in the absence of monkey serum) at the same dilution, multiplied by 100. Thus, values < 100% indicate additional killing beyond that mediated by complement alone, whereas values near 100% indicate no detectable added bactericidal effect.

### 2.9. Flow-Cytometric Evaluation of Antigen Presentation and Co-Stimulatory Marker Expression

**Figure 2:**
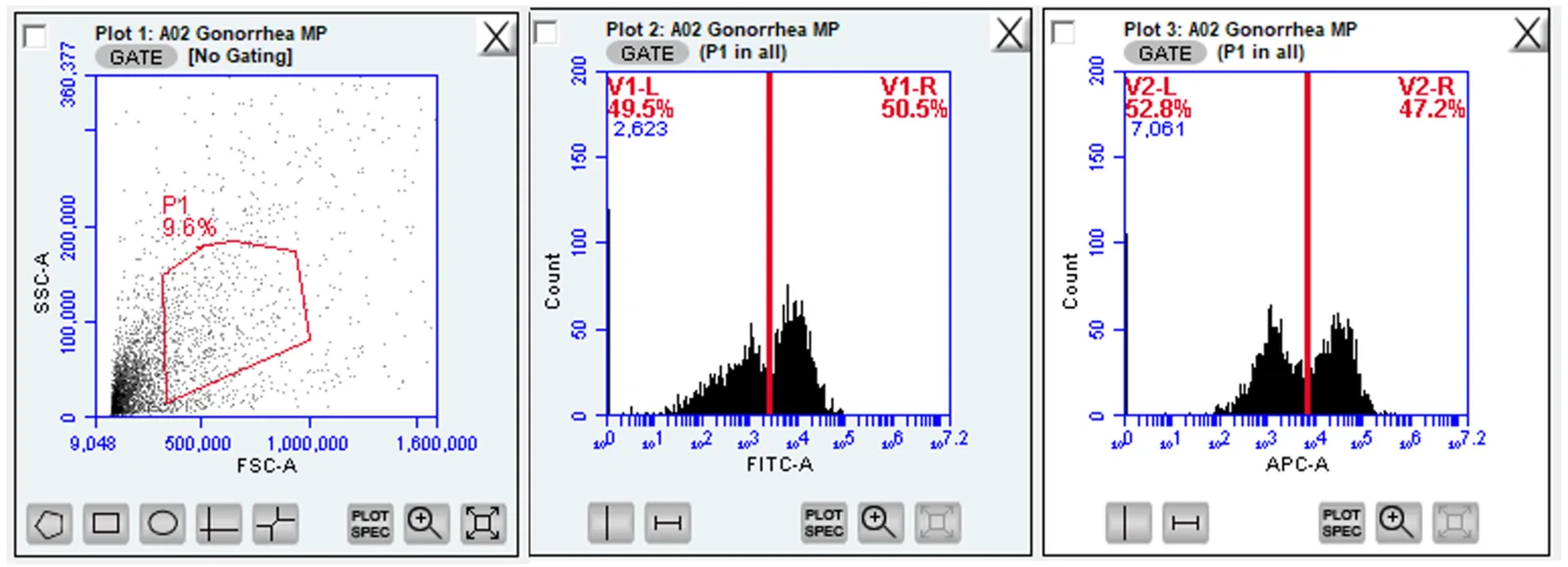
Flow-cytometric gating strategy. Cells were first identified on a forward-scatter area (FSC-A) versus side-scatter area (SSC-A) plot to exclude debris and non-cellular events. The P1 gate, encompassing the principal cell population, was applied to subsequent fluorescence analyses. Representative histograms show FITC-A and APC-A signal distributions within the P1-gated population; vertical gates delineate negative and positive event populations.

In order to assess dendritic cell activation in response to adjuvanted vaccine microparticle formulations, flow cytometry was performed using the DC2.4 cell line as a standardized in vitro model. Dendritic cells were seeded at a density of 0.01 × 10^6^ cells per well in 96-well plates and incubated at 37 °C for 24 h to allow adherence. Cells were then stimulated with 100 µg of *N. gonorrhoeae*-loaded microparticles in the presence of 50 µg of adjuvant microparticles (Delta inulin MP, Aluminum phosphate MP, or Aluminum Hydroxide MP) and incubated for an additional 24 h at 37 °C. A cell-only group was left to serve as a control. Following stimulation, cells were thoroughly washed to remove unbound particles and stained with APC- and FITC-conjugated antibodies against MHC I and MHC II, respectively (eBioscience, San Diego, CA, USA). After washing to eliminate excess antibody, fluorescence intensity was measured using a BD Accuri C6 flow cytometer (Becton, Dickinson and Company, San Diego, CA, USA). The same experimental setup and control were used to quantify the surface expression of co-stimulatory molecules, CD40 and CD80 (BioLegend, San Diego, CA, USA). Events corresponding to the main cellular population were identified using an FSC-A versus SSC-A gate (P1) to exclude debris and non-cellular events. Fluorescence distributions and positive populations were subsequently assessed within the P1-gated population using FITC-A and APC-A channels, as illustrated in Figure 2.

### 2.10. Statistical Analysis

Data are presented as mean ± standard deviation (SD) unless otherwise specified. Given the small group size (*n* = 3 per group), data distributions were assessed using individual data points and residual plots. For comparisons among multiple independent groups at a single time point (e.g., dendritic-cell activation assays), one-way ANOVA with Tukey’s post hoc test was used as a parametric approach. For longitudinal antibody responses, two-way repeated-measures ANOVA with appropriate multiple-comparison testing was applied to evaluate differences between treatment groups over time. These analyses should be interpreted as exploratory, recognizing that the limited sample size reduces the power to detect modest effects and may affect the robustness of normality assumptions. For the serum bactericidal assay, percent survival curves were summarized as mean ± SD for each group and dilution without formal hypothesis testing and were interpreted descriptively to compare overall patterns between treatment groups. Mean values ± standard deviation and *p*-values were determined individually for all experiments with GraphPad Prism 11.0.1 software.

## 3. Results

### 3.1. Characterization of the Microparticles

Spray-dried GC–MPs and separately prepared adjuvant formulations were in the low-micrometer size range. The GC-MP formulation had a mean diameter of 5.1 ± 0.215 µm, whereas delta inulin, aluminum phosphate, and aluminum hydroxide measured 3.10 ± 2.09 µm, 2.22 ± 0.43 µm, and 5.80 ± 0.82 µm, respectively (Table 2). All preparations displayed a net negative surface charge, with the GC-MP showing a zeta potential of −38.7 ± 32.7 mV, delta inulin −39.73 ± 19.7 mV, aluminum phosphate −18.2 ± 10.8 mV, and aluminum hydroxide −38.0 ± 20.1 mV. The GC-MP formulation showed a moderately broad but unimodal size distribution (PDI 0.41), while the adjuvant particles exhibited higher polydispersity (PDI 0.74–0.78), indicating more heterogeneous size distributions for the adjuvant preparations. Notably, the GC-MP formulation exhibited a relatively large standard deviation in zeta potential, reflecting variability among replicate electrophoretic mobility measurements of these micron-sized, spray-dried particles at low suspension concentrations, which are prone to partial aggregation and sedimentation. Under all measurement conditions, however, GC-MPs retained a net negative surface charge, so the zeta potential values are best interpreted qualitatively rather than as a precise quantitative descriptor of surface charge density, given the proof-of-concept nature of this study.

### 3.2. Physical Properties of N. gonorrhoeae Microparticles and Microneedles

**Figure 3:**
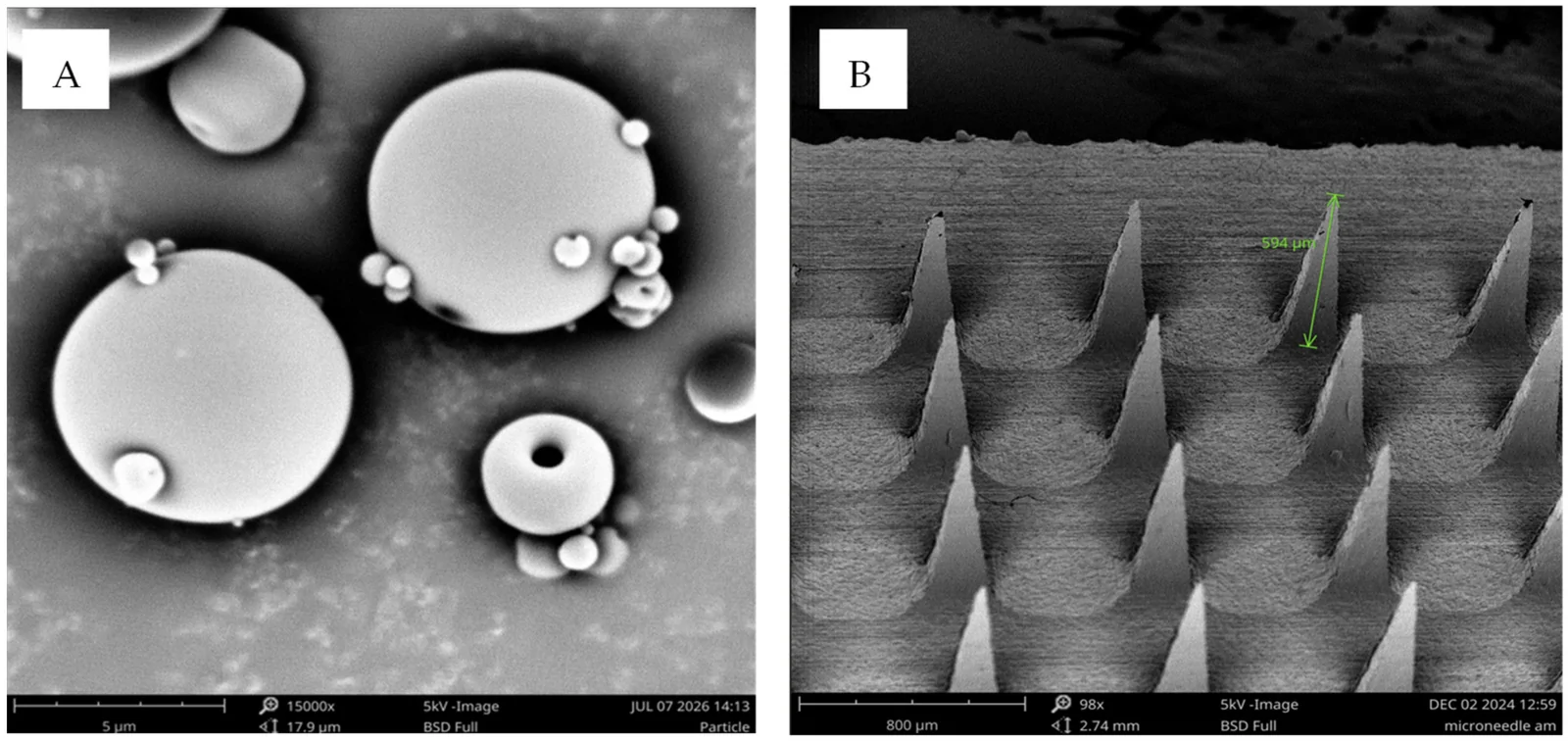
Scanning electron micrographs of *N. gonorrhoeae*-loaded microparticles (**A**) and dissolving microneedle array (**B**). Microparticles appear generally smooth and approximately spherical, but often exhibit a dimpled or donut-like morphology and are attached to satellite particles. The microneedle patch exhibits a regular array of conical needles, each approximately 594 µm in height.

Scanning Electron Microscopy (SEM) analysis showed that the *N. gonorrhoeae*-loaded microparticles were generally smooth and approximately spherical but often exhibited a dimpled or donut-like morphology, consistent with partial core collapse during drying, with occasional smaller satellite particles adherent to their surfaces (Figure 3A). The dissolving microneedle arrays exhibited regularly spaced, conical needles with uniform geometry and intact tips, with an average needle height of approximately 594 µm as indicated in the SEM images (Figure 3B), confirming the structural integrity of the patch prior to use.

### 3.3. Assessment of Humoral Antibody Response

Microneedles loaded with the gonococcal microparticles and each adjuvant were applied to the skin to deliver the vaccine and assess the humoral response. The immunization study was carried out over six weeks in *Rhesus macaques*. The following groups were used in the study: (A) Naïve, (B) Blank HSA, (C) GC MP with Advax delta inulin adjuvant, (D) GC MP with aluminum phosphate, and (E) GC MP with aluminum hydroxide. One prime dose and one booster dose were administered, and blood samples were collected just before each dose.

**Figure 4:**
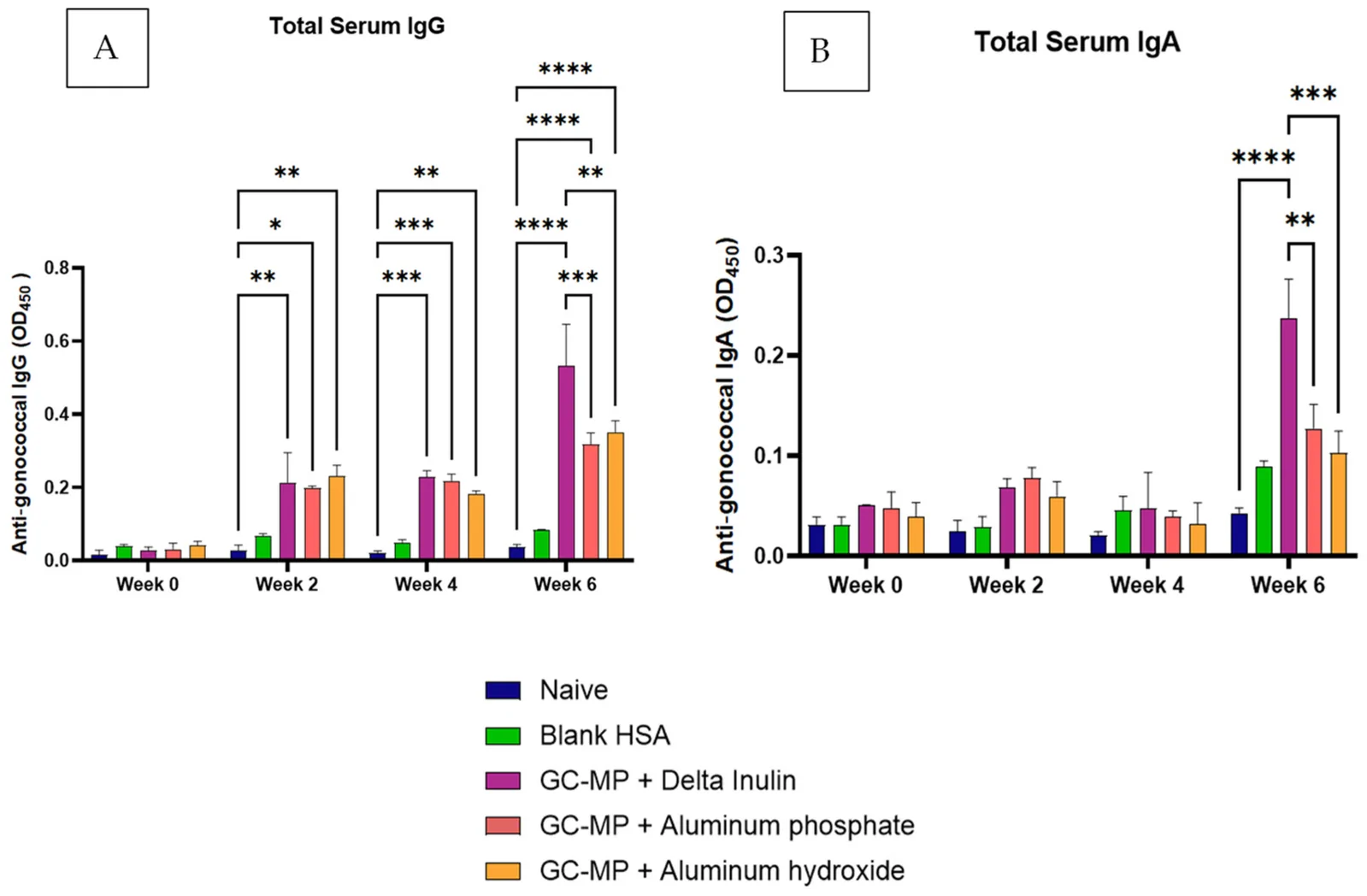
Serum anti-gonococcal antibody responses. (**A**) Total IgG. (**B**) Total IgA. ELISA conditions were identical except for the isotype-specific secondary antibody (anti-monkey IgG vs. anti-monkey IgA). Serum was tested at 1:320 dilution. Data are mean ± SD (*n* = 3 monkeys per group). Two-way repeated-measures ANOVA with multiple-comparisons testing was used as indicated, * *p* < 0.05, ** *p* < 0.01, *** *p* < 0.001, **** *p* < 0.0001.

Anti-gonococcal serum IgG OD 450 values were low at baseline in all groups (Figure 4A). Following the prime immunization, the adjuvanted GC-MP groups demonstrated increased IgG reactivity at week 2 relative to the naïve and blank HSA controls. The response was sustained and became more pronounced following the week-2 booster. At week 4, the delta inulin- and aluminum phosphate-formulated GC-MP microneedle groups exhibited significantly higher anti-gonococcal IgG reactivity than the control groups, whereas the aluminum hydroxide formulation generated a lower response. By week 6, the delta inulin formulation produced the highest mean anti-gonococcal IgG OD450 value, followed by aluminum phosphate and aluminum hydroxide. Each adjuvanted GC-MP formulation demonstrated significantly increased IgG reactivity relative to the control groups, as indicated in Figure 3A. Overall, these results show that delivery of adjuvanted GC-MP formulations by microneedle patch elicited antigen-specific serum IgG responses, with delta inulin generating the strongest post-boost response.

Anti-gonococcal serum IgA OD 450 values remained low in the naïve and blank HSA control groups throughout the study (Figure 4B). IgA reactivity in the vaccinated groups was also low through week 4, with no statistically significant group differences indicated at these earlier time points. However, a marked increase in anti-gonococcal serum IgA was observed at week 6 in all adjuvanted GC-MP groups. At week 6, the delta inulin formulation induced the highest mean anti-gonococcal IgA OD 450 value, followed by aluminum phosphate and aluminum hydroxide (Figure 4B). These findings demonstrate that the adjuvanted GC-MP microneedle formulations induced a delayed but measurable post-boost serum IgA response, with delta inulin producing the greatest response under the conditions tested.

### 3.4. Serum Bactericidal Assay

**Figure 5:**
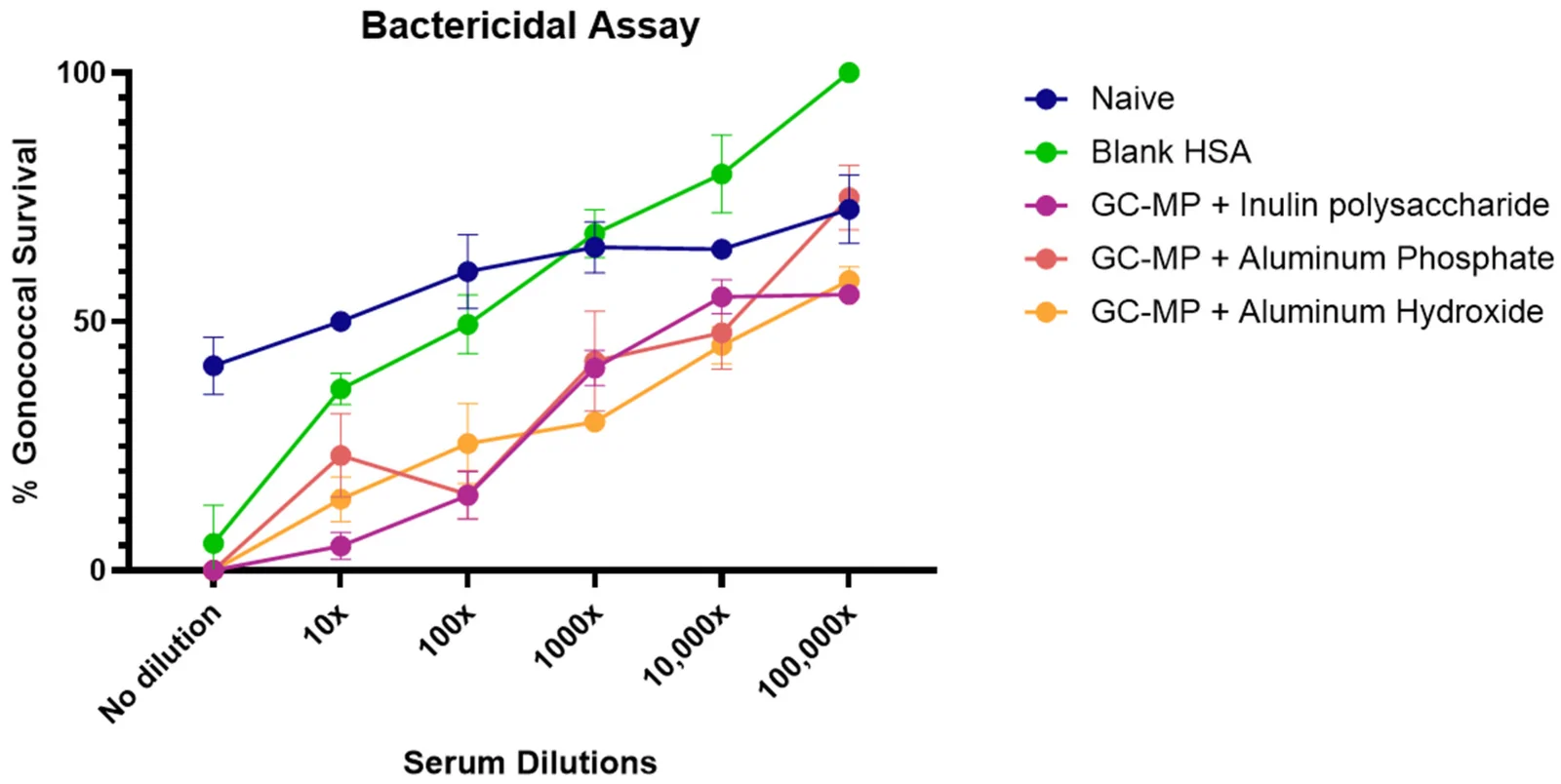
Serum Bactericidal Assay. The percent survival of the live gonococcal strain CDC-F62 was determined by counting viable colonies on an agar plate. Serum from the following groups of monkeys: Blank HSA, GC-MP + Delta Inulin, GC-MP + Aluminum Phosphate, and GC-MP + Aluminum Hydroxide was diluted 1:10 through 1:100,000. Survival values of >100% are reported as 100%. Data are mean ± SD (*n* = 3). Survival is normalized to complement-only controls. Percent survival was calculated relative to the mean CFU count in complement-only control wells at each dilution; occasional values slightly >100% (reflecting normal assay variability around the control mean) were truncated to 100% for plotting and indicate absence of detectable bactericidal activity.

The monkey sera collected at week 6 post-immunization were used to determine the serum bactericidal assay (SBA). The sera were diluted from 1:10 through 1:100,000. Significant bactericidal activity was defined as greater than 50% inhibition of bacterial growth compared with the control [23]. Serum bactericidal activity was assessed in each experimental group by measuring the percent survival of live gonococci at each dilution (Figure 5). Despite appreciable baseline killing by pooled normal human serum used as the complement source, sera from all three adjuvanted GC-MP groups showed markedly reduced gonococcal survival compared with naïve and blank HSA controls across multiple serum dilutions, including at dilutions ≥1:1000, indicating the presence of vaccine-induced bactericidal antibodies under these assay conditions.

### 3.5. Flow Cytometric Analysis of Antigen-Presenting and Co-Stimulatory Marker Expression on Dendritic Cells

**Figure 6:**
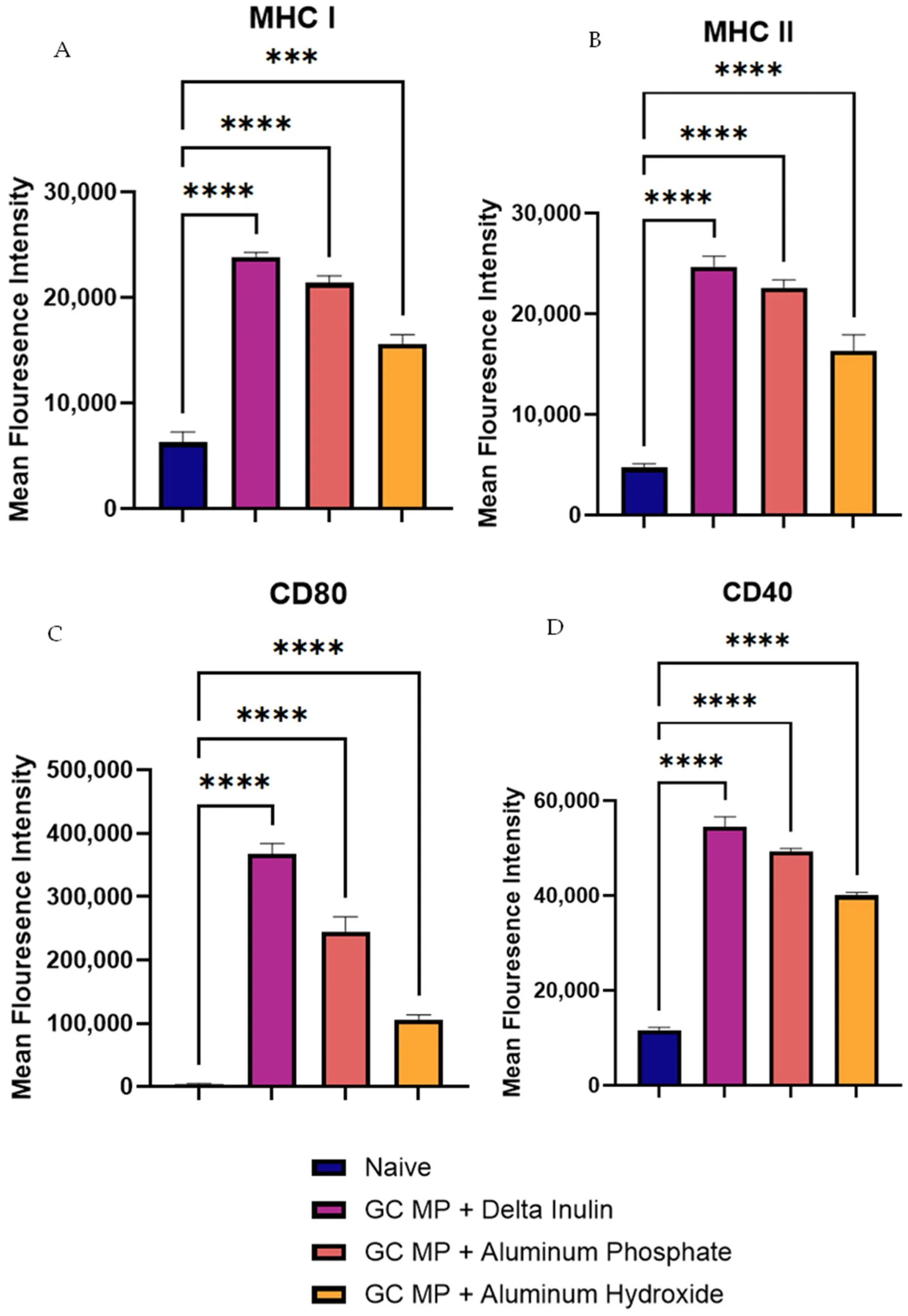
Upregulation of antigen-presentation and co-stimulatory markers on dendritic cells following stimulation with adjuvanted gonococcal microparticles. Murine DC2.4 dendritic cells were stimulated in vitro with *N. gonorrhoeae*-loaded microparticles (GC MP) formulated with delta inulin, aluminum phosphate, or aluminum hydroxide. Surface expression of (**A**) MHC I, (**B**) MHC II, (**C**) CD80, and (**D**) CD40 was quantified by flow cytometry. Data are expressed as mean ± SD, one-way ANOVA. *** *p* < 0.001, **** *p* < 0.0001.

We next assessed the ability of the various adjuvanted vaccine microparticulate formulations to activate dendritic cells. Stimulation of dendritic cells with *N. gonorrhoeae* GC-MP microparticles formulated with either of the three adjuvants significantly increased the surface expression of MHC I and MHC II compared to controls (Figure 6). Cells treated with the delta-inulin-adjuvanted GC-MP formulation exhibited the greatest upregulation of both MHC I and II molecules, followed by aluminum phosphate and then aluminum hydroxide formulations. A similar pattern was observed for the co-stimulatory molecules CD80 and CD40, in which all adjuvanted groups showed robust, statistically significant increases over naïve controls, with the delta inulin-containing formulation again associated with the highest CD80 and CD40 expression levels. Collectively, these data demonstrate that *N. gonorrhoeae* microparticles, particularly when combined with delta inulin, increased DC2.4 surface expression of antigen-presentation and co-stimulatory markers.

## 4. Discussion

This study demonstrates that an adjuvanted formalin-inactivated *Neisseria gonorrhoeae* whole-cell vaccine, encapsulated in HSA microparticles and delivered via dissolvable microneedles, can induce systemic functional antibody responses in *Rhesus macaques*. Across all three adjuvanted formulations, GC MP vaccination elicited significant increases in serum anti-gonococcal IgG and IgA, indicating that the combination of particulate GC antigen and adjuvant was immunogenic in this non-human primate model. Serum from vaccinated primates demonstrated clear complement-dependent bactericidal activity against the immunizing strain, with adjuvanted GC-MP groups demonstrating substantially lower bacterial survival across multiple serum dilutions than naïve and blank microneedle controls. This interpretation should be qualified by the fact that the pooled normal human serum used as an exogenous complement source exhibited appreciable intrinsic background bactericidal activity against CDC F62, constraining the dynamic range of the assay and likely underestimating the absolute magnitude of vaccine-induced killing; these SBA data are therefore best interpreted in terms of relative differences between vaccinated and control groups. Also, serum bactericidal activity is an established correlate of protection for *Neisseria meningitidis* and serves as an important functional readout of antibody activity; however, a definitive correlate of protection for *N. gonorrhoeae* has not yet been defined, so the SBA data presented here should be interpreted as evidence of functional antibody activity rather than as a validated correlate of protection.

These findings build on earlier murine work with a similar GP dissolvable microneedle platform, demonstrating that key features of the platform translate to primates. The low-micrometer, negatively charged GC-MPs fall within a size range expected to favor uptake by antigen-presenting cells, and the dissolving microneedles provided a practical, minimally invasive route for transdermal delivery [24,25,26]. The physicochemical properties of particulate vaccines, including size, morphology, and surface charge, are key determinants of their in vivo interactions and immunogenicity. Our GC-MPs and adjuvant microparticles fell within the low-micrometer size range (approximately 2–6 µm) with net negative zeta potentials, a profile compatible with efficient phagocytic uptake by dendritic cells and macrophages and with retention at the skin vaccination site rather than rapid systemic dispersion. The moderately broader PDI values observed for the adjuvant microparticles (PDI~0.74–0.78) suggest a heterogeneous population encompassing both smaller particles that may drain into local lymphatics and larger particles that remain at the application site, potentially providing a combination of depot effect and direct interaction with skin-resident antigen-presenting cells. Such size and charge characteristics have been associated with enhanced antigen presentation, upregulation of co-stimulatory molecules, and robust antibody responses in other particulate vaccine platforms, which is consistent with the significant DC maturation and elevated systemic IgG/IgA titers elicited by all three adjuvanted GC-MP formulations in this study [27,28,29,30,31].

The kinetics of the humoral response, with progressive increases in IgG by week 2 and a rise in IgA by week 6, suggest ongoing B-cell activation and class switching following the prime and boost regimen. Given that *N. gonorrhoeae* is a mucosal pathogen, the induction of a systemic IgA is encouraging and warrants further investigation of mucosal antibody responses in genital tract secretions.

In this study, antigen-specific serum IgG was assessed by indirect ELISA at a fixed serum dilution of 1:320 using formalin-inactivated whole-cell *N. gonorrhoeae* as the capture antigen. This assay format provided clear separation of anti-gonococcal IgG reactivity between vaccinated animals and naïve or blank HSA control groups, particularly at the post-boost time points. Because the ELISA was performed at a single fixed serum dilution, the resulting OD 450 values should be interpreted as a comparative measure of antigen-specific binding reactivity rather than as endpoint antibody titers or absolute antibody concentrations. In contrast, the serum bactericidal assay evaluated the functional capacity of week-6 sera to reduce survival of live *N. gonorrhoeae* CDC F62 in the presence of an exogenous human complement source. This assay reflects several antibody characteristics not captured by a fixed-dilution binding ELISA, including recognition of surface-accessible epitopes on intact bacteria, antibody affinity and avidity, isotype/subclass composition, epitope specificity, and the ability to activate complement. Thus, differences in the magnitude or dilution profile of ELISA reactivity and bactericidal activity are not unexpected. The use of formalin-inactivated whole-cell antigen in the ELISA may also alter the accessibility or conformation of some epitopes relative to live bacteria; however, this possibility was not directly tested in the present study [32,33]. Future experiments using matched live-cell, native outer-membrane, or defined-antigen binding assays will help determine how antigen preparation influences the relationship between ELISA reactivity and functional bactericidal activity.

In addition to the humoral and bactericidal responses observed in vaccinated macaques, the dendritic cell activation profile elicited by the adjuvanted gonococcal microparticles provides important mechanistic insight into how this platform may overcome the well-described immune evasion strategies of *N. gonorrhoeae* [34]. All three adjuvanted GC-MP formulations significantly upregulated surface MHC I and MHC II expression on dendritic cells, suggesting the ability to enhance antigen-processing and presentation through both class I and class II pathways. Concurrent increases in the surface expression of the co-stimulatory molecules CD40 and CD80 on dendritic cells further suggest that these antigen-presenting cells acquire a more mature, immunostimulatory phenotype capable of providing help for T-cell priming and B-cell help [35,36]. These experiments, performed in the murine DC2.4 dendritic cell line, were intended to compare the relative effects of the different adjuvanted GC-MP formulations on DC activation under controlled conditions, rather than to directly recapitulate primate DC responses. Notably, the delta-inulin-adjuvanted GC-MP formulation induced the highest levels of MHC I, MHC II, CD40, and CD80, consistent with strong dendritic cell activation. This is consistent with the finding that delta inulin is an agonist of DC-SIGN, a receptor expressed almost exclusively on dendritic cells and some monocytes [18,37]. This could potentially explain the favorable induction of class-switched, high-affinity antibody responses. Together, these findings imply that the observed serum IgG/IgA and bactericidal activity reflect underlying enhancement of both antigen presentation and co-stimulatory signaling. This highlights the potential importance of adjuvant selection for improving the quality of adaptive immunity generated by this microneedle patch platform.

While all three adjuvants, delta inulin, aluminum phosphate, and aluminum hydroxide, were associated with the induction of anti-gonococcal IgG and IgA plus bactericidal activity, specific adjuvants may still have unique benefits when incorporated into microneedle patches. In particular, aluminum phosphate and aluminum hydroxide adjuvants are known to be highly Th2-biased and fail to induce cytotoxic CD8 T cell responses needed for killing of intracellular bacteria [38]. By contrast, delta-inulin adjuvant has been shown to be a potent inducer of both Th2 and Th1 memory responses, including the induction of memory CD8 T cell responses [39]. It does so via effects on dendritic cells via DC-SIGN [18], resulting in upregulation of MHC and co-stimulatory molecules, as we observed in vitro. This is independent of the inflammasome pathways utilized by alum adjuvants that are associated with immune cell death and reactogenicity [40]. Delta-inulin adjuvant has been shown to enhance vaccine efficacy against a broad range of viral and bacterial pathogens where both humoral and cellular immunity are important for control of the pathogen, including anthrax [41], listeria [42], and M. tuberculosis [43], amongst others. By contrast, aluminum phosphate and aluminum hydroxide are thought to act through a combination of antigen depot effects, particulate uptake by phagocytes, and activation of innate pathways, including NLRP3 inflammasome signaling, which biases responses toward Th2-skewed antibody production with more limited effects on cellular immunity [44,45]. With its ability to induce complement-activating serum antibodies and strong T cell memory responses, delta inulin may surpass alum-based adjuvants in microneedle platforms by more directly targeting dendritic cells and promoting the high-quality bactericidal antibody and T cell responses needed for effective gonococcal immunity.

Particle characterization revealed relatively high polydispersity indices for the adjuvant microparticles, indicating heterogeneous size distributions that could impact cellular uptake and adjuvant function [46,47]. Further formulation work to narrow these distributions and to more directly link particle properties to immunological outcomes may be informative.

Several limitations should be acknowledged. This study was designed as a proof-of-concept evaluation of immunogenicity and bactericidal activity rather than as a definitive preclinical safety and efficacy study. The number of animals per group was modest, a limitation of NHP studies, which limited statistical power and the ability to detect differences between the adjuvants. The formalin-inactivated whole-cell *N. gonorrhoeae* antigen used in this GC-MP microneedle platform presents a wide array of native surface and periplasmic antigens, which is advantageous at a proof-of-concept stage when the precise protective targets are not fully defined. At the same time, historically whole-cell gonococcal vaccines have generally provided strain- or serogroup-restricted protection in humans, and antibodies directed against highly immunogenic components such as reduction-modifiable protein (Rmp) can block complement-dependent killing by otherwise bactericidal antibodies. In this primate study, we assessed serum bactericidal activity only against the homologous CDC F62 strain and did not dissect antibody specificity against individual antigens such as Rmp. As a result, the breadth of strain coverage across diverse Por types and the functional impact of any anti-Rmp antibodies remain undefined and should be considered when interpreting the present findings. Although serum volume was sufficient for the analyses reported here, the present proof-of-concept study was not designed or powered to comprehensively assess antibody breadth across a diverse panel of *N. gonorrhoeae* isolates. We evaluated functional bactericidal activity only against CDC F62 and measured antigen-specific ELISA reactivity using formalin-inactivated whole-cell antigen prepared from the vaccine strain. Consequently, the breadth of binding and functional antibody responses against heterologous gonococcal strains remains unknown and should be addressed in future studies using a standardized panel of clinically relevant isolates. A GC antigen–loaded microneedle patch without adjuvant was not included in this primate study, so the absolute effect size attributable to the adjuvants relative to an unadjuvanted GC-MP microneedle formulation cannot be quantified here. The observed differences among the delta-inulin, aluminum phosphate, and aluminum hydroxide groups should be interpreted as exploratory comparisons among complete formulations rather than quantitative measures of individual adjuvant effects. Future NHP studies should include an unadjuvanted GC-MP microneedle group and, ideally, sufficient group sizes and dose-ranging arms to determine the incremental effect and optimal dose of each adjuvant. Although animals were monitored clinically throughout the study and no vaccine-related adverse events or notable local skin reactions were observed following microneedle application, this work was not designed as a formal toxicology study and did not include detailed clinical pathology or histopathology, so conclusions regarding biosafety remain preliminary. A limitation of the in vitro DC maturation assay is that we did not include a canonical positive control such as LPS and therefore cannot directly compare the magnitude of activation elicited by the vaccine formulations to that induced by a strong TLR agonist. Future studies will incorporate such positive controls to further benchmark the quality and intensity of dendritic cell activation. In vitro dendritic-cell activation was evaluated using the murine DC2.4 cell line, which offers a standardized and reproducible model for comparing formulations but may not fully capture species-specific features of rhesus macaque dendritic-cell biology; accordingly, these mechanistic data should be interpreted as supportive rather than as a direct surrogate for primate DC responses. Another limitation was that the current study focused on systemic humoral responses and did not assess T-cell immunity or local genital mucosal responses, both of which are likely to be important for protection against gonococcal infection and for clearance of established colonization. The antigen was derived from a single *N. gonorrhoeae* strain, and bactericidal activity was assessed against a limited strain background. Given the extensive antigenic and phase variation characteristic of *N. gonorrhoeae*, future studies should better evaluate the breadth of strain coverage. Although the serum bactericidal assay is a strong functional readout, the study did not include an in vivo pathogen challenge, so direct evidence of the microneedle vaccine patches’ ability to induce in vivo protection in a primate species remains unknown. Although 450 µg of each adjuvant was used to enable an initial comparison at the same nominal mass, equal masses of delta-inulin, aluminum phosphate, and aluminum hydroxide should not be considered biologically equivalent doses. Adjuvant activity depends on composition, particle characteristics, interactions with the antigen, delivery route, and the fraction released from the microneedle matrix. Furthermore, clinical experience with delta-inulin has largely involved milligram quantities in liquid formulations administered intramuscularly, which cannot be directly extrapolated to cutaneous delivery via dissolving microneedles. Future studies should therefore evaluate multiple delta-inulin doses, quantify patch loading and the dose delivered into the skin, and assess local and systemic safety to define the minimum effective and optimal doses for this platform. In addition, although we fabricated microneedle patches using a standardized protocol developed in our previous work and checked gross structural integrity and loading, we did not formally evaluate inter-batch reproducibility in this proof-of-concept NHP study; therefore, conclusions about manufacturing robustness are limited. We also did not systematically evaluate local tolerability using predefined scores for erythema, edema, induration, ulceration, or other application-site findings, and we did not obtain standardized photographs or skin histopathology. Although routine monitoring did not identify clinically notable application-site reactions requiring veterinary intervention, mild or transient reactions may not have been captured consistently. Accordingly, this study does not provide a definitive local safety assessment of the microneedle formulations. Future studies should include prospective application-site scoring at defined intervals, standardized photography, clinical pathology, and, where appropriate, histopathological examination.

Despite these constraints, the present data provide meaningful proof of concept that an adjuvanted whole-cell inactivated gonococcal vaccine delivered via dissolving microneedles can generate class-switched bactericidal antibodies in a relevant large-animal model. This supports further preclinical development focused on optimizing adjuvant formulations, characterizing mucosal and cellular immunity, and testing cross-protective efficacy against diverse clinical isolates.

## 5. Conclusions

The present study demonstrates that formalin-inactivated GC-MP vaccines formulated with delta-inulin, aluminum phosphate, or aluminum hydroxide and delivered using dissolving microneedles were immunogenic in *Rhesus macaques* and generated sera with measurable bactericidal activity against the homologous gonococcal strain. Among the formulations tested, the delta-inulin-containing formulation produced the highest mean post-boost antibody responses under the conditions examined. However, because we did not include an unadjuvanted GC-MP group, we cannot determine the incremental contribution of each adjuvant. Further studies incorporating a GC-MP-only comparator, larger groups, adjuvant dose-ranging, and broader functional endpoints are needed before drawing conclusions about adjuvant enhancement or superiority.

## Figures and Tables

**Table 1:** In vivo study groups.

Group	Loaded Antigen	Adjuvant	Dose
A	Naïve	N/A	N/A
B	Human serum albumin	N/A	950 μg of Human Serum Albumin
C	GC MP	Advax^®^ (delta inulin)	950 μg of GC MP + 450 μg of Advax^®^ (delta inulin)
D	GC MP	Aluminum phosphate	950 μg of GC MP + 450 μg of Aluminum phosphate
E	GC MP	Aluminum Hydroxide	950 μg of GC MP + 450 μg of Aluminum Hydroxide

**Table 2:** Characterization of gonococci vaccine and adjuvant microparticles.

	GC-MP (Gonococcal Microparticle)	Delta Inulin Microparticles	Aluminum Phosphate Microparticles	Aluminum Hydroxide Microparticles
**Particle Size (µm)**	5.1 ± 0.215	3.098 ± 2.090	2.218 ± 0.433	5.798 ± 0.822
**Zeta Potential (mV)**	−38.7 ± 32.7	−39.73 ± 19.7	−18.2 ± 10.8	−38.0 ± 20.1
**Polydispersity Index**	0.41	0.74	0.78	0.75

## Data Availability

Data will be available on request.
